# Dr. Baillie

**Published:** 1824-07

**Authors:** 


*Monument to* Dr. Baillie.—We understand that a meeting of the subscribers for the intended monument to the memory of this distinguished physician, is to be held at the Freemason's Tavern, on the 1st of July. We regret exceedingly that our limits will not permit us to do more than express our sincere wish that the object may succeed in a manner deserving alike of the late eminent individual and of the profession, to which he was so long an ornament.

